# El Niño Southern Oscillation and Leptospirosis Outbreaks in New Caledonia

**DOI:** 10.1371/journal.pntd.0002798

**Published:** 2014-04-17

**Authors:** Daniel Weinberger, Noémie Baroux, Jean-Paul Grangeon, Albert I. Ko, Cyrille Goarant

**Affiliations:** 1 Yale School of Public Health, New Haven, Connecticut, United States of America; 2 Institut Pasteur, Institut Pasteur International Network, Noumea, New Caledonia; 3 Direction des Affaires Sanitaires et Sociales de la Nouvelle-Calédonie, Noumea, New Caledonia; 4 Yale School of Medicine, New Haven, Connecticut, United States of America; 5 Oswaldo Cruz Foundation, Salvador, Bahia, Brazil; University of Tennessee, United States of America

## Abstract

Leptospirosis is an important cause of seasonal outbreaks in New Caledonia and the tropics. Using time series derived from high-quality laboratory-based surveillance from 2000–2012, we evaluated whether climatic factors, including El Niño Southern Oscillation (ENSO) and meteorological conditions allow for the prediction of leptospirosis outbreaks in New Caledonia. We found that La Niña periods are associated with high rainfall, and both of these factors were in turn, temporally associated with outbreaks of leptospirosis. The sea surface temperature in El Niño Box 4 allowed forecasting of leptospirosis outbreaks four months into the future, a time lag allowing public health authorities to increase preparedness. To our knowledge, our observations in New Caledonia are the first demonstration that ENSO has a strong association with leptospirosis. This association should be tested in other regions in the South Pacific, Asia or Latin America where ENSO may drive climate variability and the risk for leptospirosis outbreaks.

## Author Summary

The El Niño Southern Oscillation is a major ocean – atmosphere phenomenon that strongly contributes to the timing and intensity of rainfall in the tropical Pacific islands and beyond. As a consequence, it also has a major effect on the number of cases of leptospirosis. By incorporating oceanographic parameters in models, we have been able to predict leptospirosis outbreaks in New Caledonia 4 months in advance. We discuss that this forecasting delay might be used to implement timely interventions prior to the occurrence of outbreaks. Possible interventions include controlling rodent reservoir populations before population growth and maintaining sewage networks or river banks. The major impact of El Niño on the climate of Pacific Islands Countries and Territories suggests that other countries might be able to build similar predictive models.

## Introduction

Leptospirosis is an important zoonotic disease with high incidence in tropical and subtropical regions worldwide, with more than 850,000 cases a year, a high incidence in tropical regions worldwide [1] and a case fatality ratio frequently exceeding 10% [2]. It has also been reported as an emerging or re-emerging disease, including in temperate countries [3], [4]. More than 230 pathogenic serovars have been described, belonging to 9 pathogenic and 5 intermediate *Leptospira* species [5]. Humans acquire infection through direct contact with the urine or kidney tissues of infected reservoir mammals or, most frequently, through contaminated water [6], [7].

Because of this transmission routes, climate is a major driver of leptospirosis. Increased rainfall leads to increased human exposure [8] through both increased survival of the bacteria in the environment and increased exposure of humans to surface water [9]. Extreme climatic events and floods have frequently been associated with leptospirosis outbreaks [10]–[12]. Rainfall also leads to larger rodent populations, further contributing to increased environmental contamination [13].

El Niño events occur when a warm ocean water pool occasionally reaches the Pacific coast of Latin America. The opposite phase is called “La Niña” and refers to the movement of this warm ocean water pool westwards. This oceanic phenomenon is strongly linked to atmospheric pressure changes known as the “Southern Oscillation,” and their interplay is usually called El Niño Southern Oscillation or ENSO. This interaction between ocean temperatures and pressure is associated with increased rainfall, including heavy rain episodes and floods. Conversely, droughts can affect the opposite side of the South Pacific (e.g. Australia) during El Niño episodes. This phenomenon is quasi-periodic and occurs every two to seven years.

Because ENSO is an important determinant of year-to-year variability in weather, including heavy rainfall and drought, a number of studies have focused on its impact on transmission and disease patterns. ENSO has been associated with outbreaks of cholera and a range of vector-borne diseases including leishmaniasis, malaria, and arboviral diseases in various countries of Latin America and Asia [14], [15]. More recently, together with rodent density, ENSO has also been associated with hantaviruses incidence in China [16]. Surprisingly, despite its major impact on rainfall, investigations have not clearly demonstrated a link between ENSO and leptospirosis, though it has been suspected to be related to a four-fold increase in the incidence of leptospirosis in Guadeloupe from 2002 to 2004 [17].

New Caledonia, a French territory in the Southwest Pacific (20–22°S, 164–167°E), provides a unique setting to understand the epidemiology of leptospirosis, a major public health concern for the island [18]–[20]and its link to climatic variability. Notification of leptospirosis to the health authority has been mandatory since 1991. Furthermore surveillance is laboratory-based, where a single reference laboratory has been performing laboratory confirmation of the diagnosis since 1989 [19], [21], [22]. This system has yielded accurate data on human leptospirosis incidence, which occasionally exceeds 100 cases per 100 000 inhabitants a year. Leptospirosis cases in New Caledonia are most common among people living in tribes or villages in the rural backcountry, though it also affects a few urban citizens. The disease is strongly seasonal, with epidemics occurring during the hot and rainy season from January through June each year. However, strong variations in incidence occur between years, epidemics occurring during periods of heavy rains [23].

Although suspected [18], the link between ENSO and year-to-year variation in the size of seasonal outbreaks has not been investigated with extended time series data from New Caledonia. Furthermore an improved understanding of how climatic and meteorological factors contribute to epidemics may allow for early-warning outbreak predictions and implementation of more effective public health preparedness and response. In this study, we aimed to determine whether ENSO-related variations in climate and meteorological conditions are associated with leptospirosis epidemics in New Caledonia and to determine whether such data could be used to build an early-warning system.

## Methods

### Laboratory diagnostic techniques

For leptospirosis diagnosis, serum and/or urine (and occasionally other sources, like cerebrospinal fluid) specimens are routinely received at Institut Pasteur laboratory from all hospitals, health centers and private laboratories throughout New Caledonia. As recommended by the World Health Organization [24], serology is carried out using the reference Microscopic Agglutination Test (MAT) with a 11-strain panel suited to the local epidemiology [18]. The microbiological diagnosis relied on culture and a nested PCR targeting the 16SrRNA gene [25] prior to 2006 and then shifted to a real time PCR specific for pathogenic leptospires. From 2006 to 2011, the *lfb1* gene was targeted using SYBR Green I technology [26]; the technique was then changed in 2012 for a TaqMan probe-based technique targeting the pathogenic *Leptospira*-specific *lipL32* gene [27]. Both techniques have a similar lower limit of detection [28], a finding confirmed during our technique transition period (data not shown).

### Case definitions and data collection

The case definitions used for leptospirosis surveillance in New Caledonia have been the same since 1995 and were described previously [18]. Briefly, based on a clinical suspicion, cases were considered as confirmed if *Leptospira* is cultured, or if its genome is evidenced by PCR or real time PCR from blood, urine or cerebrospinal fluid, or if a seroconversion (from nil to at least a 400 titer) or a significant sero-ascension (at least a fourfold raise in titers) is observed in paired sera using the MAT. The case was considered as probable leptospirosis if a single MAT titer ≥800 is observed for at least one pathogenic serogroup in the serum of a patient with a clinical suspicion.

Anonymous data used in this study were extracted without patient identification from the Institut Pasteur laboratory database and originated from routine diagnostic activities as part of public health surveillance. The corresponding biobank was declared to the French Ministry of Research (DC-2010-1222, Collections number 1 and 2). We excluded patients from other Pacific Islands Countries or Territories whose specimens are sometimes submitted to Institut Pasteur laboratory. The date assigned to each case was the month when the first biological specimen was collected for submission to the laboratory (e.g. date of the first serum in the case of paired sera, even if MAT-negative).

The ENSO is studied using both atmospheric and oceanographic parameters. The Southern Oscillation Index (SOI) reflects the atmospheric pressure difference between Darwin in Australia and Tahiti in French Polynesia [29]. A sustained value of the SOI under −8 (or above +8 respectively) is considered as reflecting an El Niño (or a La Niña respectively) status of the oscillation. Oceanographic parameters include Sea Surface Temperatures (SST) and their anomalies [30] in four equatorial oceanic “Boxes”, Boxes 1 and 2 being adjacent to South American Pacific coasts and Box 4 being the most westward one. An El Niño phase of the ENSO is related to positive SST anomalies in the Niño Box 4. We studied SST in the most commonly used region 3.4 and in the Box 4, the most relevant area from a geographical point of view. Other indices have been proposed and used including the Oceanic Niño Index (ONI) [31] and the Multivariate El Niño Index (MEI) that combines sea-level pressure, components of the surface wind, SST, surface air temperature and cloudiness indicators [32], [33].

The study period was January 2000–December 2012 for leptospirosis cases and July 1999–December 2012 for oceanic and atmospheric ENSO parameters. Climatic and oceanographic data were downloaded from the Australian Bureau of Meteorology (Southern Oscillation Index, SOI) or the National Oceanic and Atmospheric Administration (NOAA) websites (Sea Surface Temperatures and anomalies, ONI and MEI). Meteorological data for New Caledonia were chosen from 3 leptospirosis hot spots located in the middle of the country [23], all kindly provided by Meteo France. A map displaying the location of the meteorological stations is provided as Figure S1 as Supporting Information.

### Statistical analysis and descriptive modeling

We first tested for an association between monthly cases of leptospirosis and each of the El Niño variables (surface temperature, El Niño indices) and climate variables (average monthly minimum/maximum temperature, cumulative rainfall over the previous 1–8 months. These associations were evaluated using negative binomial regression because the data exhibited evidence of overdispersion. All of the models included harmonic terms (sine and cosine terms that repeat every 12 months) to control for consistent, shared seasonal variations that were unrelated to variations in the climate variable. The baseline model included the harmonic terms but did not include any climate or meteorological variables. In addition to the climate and meteorological variables, we tested whether the number of cases in month t-1 or t-12 predicted the number of cases in the current month (t) (log-transformed variable, adding 0.5 to each observation). Since the effect of climate on disease might not be immediate, we evaluated associations between leptospirosis cases and each of the El Niño and climate variables with lags of 0 to 6 months. In total, 46 different variables were tested with 7 lags each (0 to 6 months) for a total of 322 single variable models. Bayesian information criteria (BIC) were used to identify the variables that best explained the disease data.

We also evaluated the correlations among the climate and meteorological variables using partial correlations (PROC CORR in SAS v9.3), controlling for harmonic variation with a sine and cosine variable with a 12 month harmonic.

### Multivariate analysis

To estimate the total contribution of climate and meteorological variation to the burden of leptospirosis, we next performed multivariate analysis. Because there was a high degree of collinearity among the potential climate and meteorological variables [34], we reduced the number of variables in the model by first performing principal components analysis (PROC PRINCOMP in SAS v9.3) [35]. The first 5 principal components accounted for 81.3% of the variation in the climate and meteorological variables (Supporting **Table S1**). We then used the BMA package in R [36], [37] to perform BIC-based model averaging [38]. The variables for model averaging included the first five principal components [35], sine and cosine terms with a 12 month period, and 1-month, and 12-month autoregressive terms (log-transformed). The outcome variable was monthly leptospirosis cases. The number of leptospirosis cases attributable to each of the principal components was determined by: predicted- (predicted/exp(βx_i_*x_i_)), which compares the total number of predicted cases in each month with the number of predicted cases when the component (βx_i_) is held to 0. The attributable percent is calculated as the number of cases attributable to each component divided by the total number of predicted cases. This value can be interpreted as the percent of cases that would not have occurred if the specific factor was held at its mean (no variation).

### Predictive modeling

We considered whether outbreaks of leptospirosis could be forecast several months in advance. The goal was to develop a simple model based on a small number of components that are available in a timely manner. Based on the univariate analyses, we decided to use sea surface temperature anomalies (box 4, lagged by 4 months) as the primary prediction variable. SST anomaly (box 4) was among the best predictors of leptospirosis cases based on BIC score and is available more rapidly than the ONI index. We also included sine and cosine terms (12 month period), and the number of cases 12-months prior to the month being forecast (log-transform). The initial training period for fitting the model was from 2000–2006, and we iteratively added an additional month onto the training period and refit the model. For each of these training periods, we fit the model and then extrapolated the number of cases 4 months after the end of the training period. The observed number of cases 4 months past the training period was compared to the predicted number of cases using correlations.

We also considered whether these models could accurately detect whether an epidemic would occur (binary for the number of cases being above or below an epidemic threshold). An epidemic threshold was estimated using the Serfling approach [39], where a harmonic baseline was fit to the square root-transformed leptospirosis data from June–September from 2000–2012. An outbreak was defined according to the forecasting model if the observed or expected number of cases exceeded the upper 95% prediction interval.

## Results

### Number of cases, fatality rate and demographic and climate features

**Figure 1:**
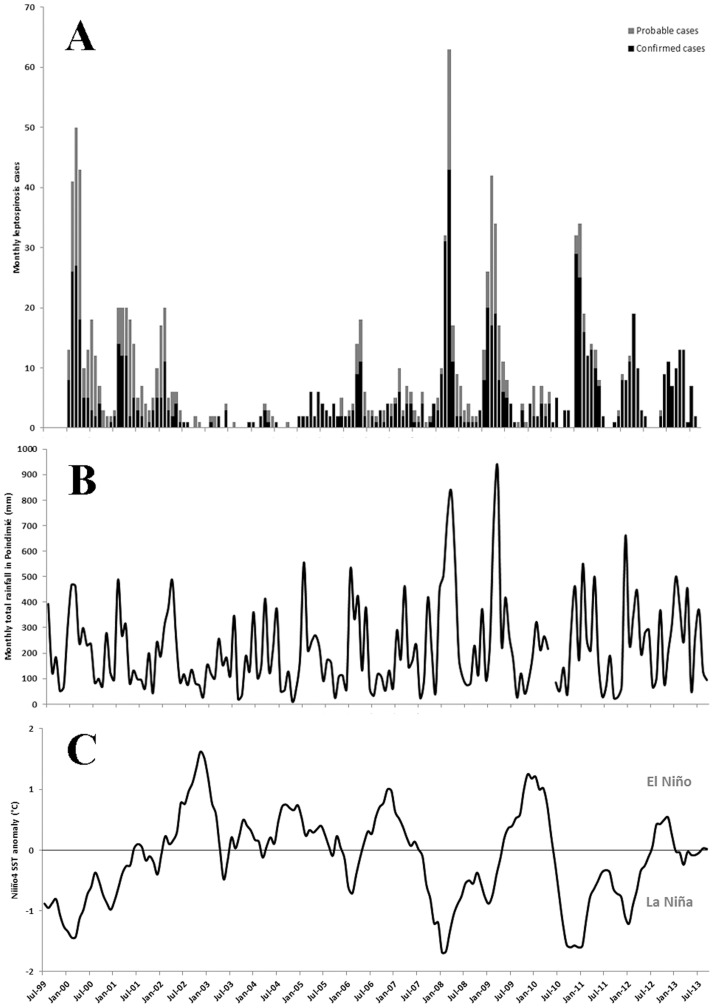
Time series of leptospirosis cases (A), monthly rainfall in Poindimie (B), and Sea Surface Temperatures anomaly in Niño Box 4 (C) for the period from 2000 to 2012 in New Caledonia.

A total of 1163 (731 confirmed and 432 probable) human leptospirosis cases were diagnosed over the period 2000–2012. The age range was 1–84.8 year old (out of 729 archived data), the median age was 34.0. Most cases were males (Sex-ratio Male/Female = 1.90 out of 1150 data available). During this same period, leptospirosis was considered by New Caledonian Health Authority as the cause for 40 deaths, varying between one (in 2007 and 2010) and 7 deaths per year (in 2001), a mean 3.4% fatality rate over the study period [40]. During the surveillance period, the mean annual incidence was 37.4 cases per 100,000 population. The number of cases however was highly variable (**Figure 1A**) and as a consequence, the incidence fluctuated between 5.6 (in 2004) and 66.4 (in 2009) cases per 100,000 population.

During our study period significant variations occurred in the SOI and SST anomalies in the oceanographic Niño Box 4 (**Figure 1C**). There were 4 El Niño and 5 La Niña periods from 2000–2012. There were moderate but significant negative associations between the El Niño indices and rainfall and temperature measurements (Supporting **Table S2**).

### Association between leptospirosis cases and climate and meteorological conditions

There was a significant association between leptospirosis cases and each of the El Niño indices, sea surface temperature anomalies, and rainfall (**Figure 1B**), even when controlling for consistent shared seasonal variations with harmonic variables (**Table 1**, Supporting **Table S2**). In particular, the Oceanic Niño Index and SST anomaly (box 4) best fit the leptospirosis data (**Table 1**, Supporting **Table S3**). The association between leptospirosis and SST anomaly was greatest with a 4 month lag (cases increased 4 months after the change in SST) (**Table S2**). The meteorological variable with the best fit to the leptospirosis data was the cumulative rainfall in Poindimie over the previous 8 months (Supporting **Table S3**).

**Table 1:** Association between monthly leptospirosis cases and El Niño and meteorological variables, controlling for 12-month harmonic variation.

Bayesian Information Criterion (BIC)	Predictor variables
831.8	Oceanic Niño Index (ONI), 3-month lag
833.5	Sea surface temp. anomaly (box 4), 4-month lag
837.8	Sea surface temp. anomaly (box 3. 4), 3-month lag
850.0	Multivariate ENSO Index (MEI), 4-month lag
859.8	Southern Oscillation Index (SOI), 4-month lag
870.7	Rainfall Poindimie, cumulative over previous 8 months
883.4	Rainfall Bourail, cumulative over previous 7 months
887.3	Rainfall Ponerihouen, cumulative over previous 7 months
890.9	Mean maximum temp. Poindimie, unlagged
893.2	Mean maximum temp. Bourail, 4 month lag
897.4	Mean maximum temp Ponerihouen, 5-month lag
898.0	Baseline model (No climate or El Niño variables)

Selected single predictor models. Smaller values of BIC signify a better fit to the data. All lags between 0 and 6 months were tested for each variable. For the cumulative sum variables, all ranges from the previous 0 to 9 months were tested. Only the lag or cumulative sum with the lowest BIC score for each variable is presented here. A difference in the BIC scores of greater than 2 is considered to be important. All of the models include sine and cosine terms with a 12-month period. For the full table of results, see Supporting **Table S3**.

**Figure 2:**
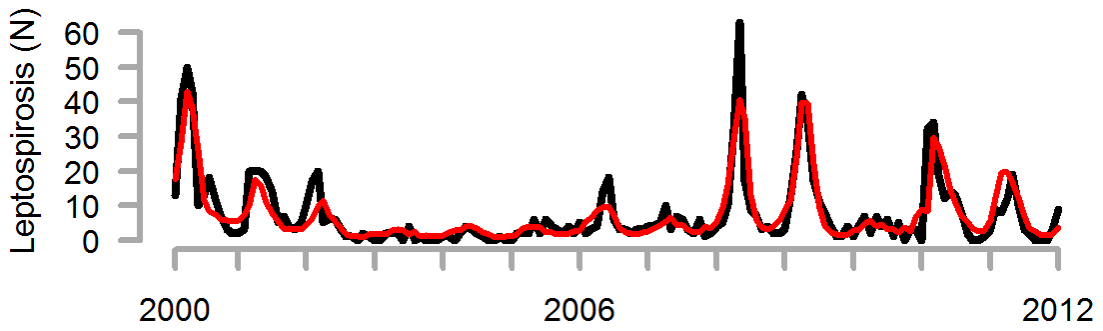
Observed (grey) and predicted (red) cases of leptospirosis occurring in each month in New Caledonia, 2000–2012.

We next used model averaging to fit a multivariate model that included the climate and meteorological variables (represented by 5 principal components), harmonic terms, and autoregressive terms. While the associations between leptospirosis and individual principal components are not meaningful in this context, the full averaged model accurately captured the dynamics of leptospirosis cases, including the large epidemics and the series of weaker years in the mid-2000s (**Figure 2**). Based on the model fit, we estimated that 35.8% of leptospirosis cases were attributable to variability in the climate and meteorological variables.

### Forecasting of leptospirosis epidemics using sea surface temperature

**Figure 3:**
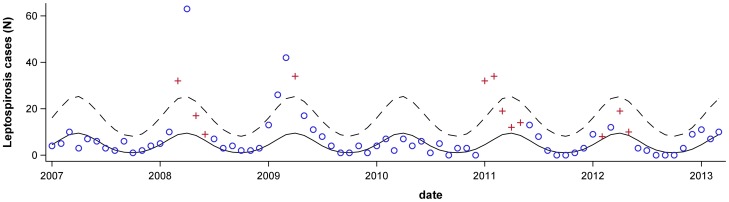
Seasonal baseline (solid line) and upper limit of the 95% confidence interval (dashed line). The observed number of leptospirosis cases in each month is shown for 2007–2012. Blue circles indicate months that had been forecasted to be below the epidemic threshold and red crosses indicate months where the forecast predicted an epidemic. When the red crosses are above the 95% confidence interval, this indicates that the forecast had correctly predicted an epidemic intensity in that month.

Finally, we considered whether we could forecast the number of leptospirosis cases four months into the future using a simple model that included SST anomaly in Niño Box 4, which was among the best predictors of leptospirosis in the single variable analysis and is available nearly in real-time. The model included the SST anomaly variable (Niño Box 4, lagged by 4 months), the number of leptospirosis lagged by 12-months and the harmonic variables to forecast the number of cases four months ahead. There was a moderate-to-strong correlation between the observed and forecasted values (Spearman's r = 0.74). The observed number of leptospirosis cases exceeded an epidemic threshold in 2008, 2009 and 2011. The model correctly predicted that the number of cases would exceed the threshold in each of these years (**Figure 3**). Likewise, the model correctly predicted the 2007 and 2010 would be non-epidemic years. The model incorrectly predicted that the observed number of cases would exceed the epidemic threshold in 2012 (**Figure 3**).

For the 2008 and 2011 epidemics, the model correctly predicted the first month that would be above the threshold (**Figure 3**). In contrast, in 2009, the first month that was predicted to be above the threshold was two months after the actual start of the epidemic. Of note, the 4-month-lagged climate variable still would have predicted an epidemic two months in advance of the actual start of the 2009 epidemic.

## Discussion

Leptospirosis is a major infectious disease in Pacific Island Countries and Territories (PICTs) [41]. It usually has a rural endemic pattern with seasonal oscillations in incidence and strong inter-annual variability in incidence. Here we demonstrate that ENSO-associated variations in SST, and related variations in rainfall and temperature, are associated with large variations in leptospirosis incidence in New Caledonia.

La Niña periods were previously shown to be responsible for heavy rainfall in New Caledonia [42], [43]. Mechanistically, there is strong support for the notion that climate can influence leptospirosis incidence. Apart from isolated extreme rainfall events, known to be possible triggers of leptospirosis outbreaks [12], [44], [45], our results suggest a more complex and longer lasting mechanism. The single variable analyses show that the accumulated rainfall during the previous 8 months is linked to leptospirosis incidence. One possible explanation is that periods of intense rainfall build-up the rodent reservoir, both by increasing the density of rodent populations and the prevalence of *Leptospira* within these populations [13], which would in turn increase environmental contamination. In the environment itself, wet and hot conditions will also favor *Leptospira* survival, also contributing to increased exposure in humans [13]. During these periods, humans are also more likely to be exposed to floods or flooded grounds, again increasing exposure risk. Similarly, by facilitating transmission between animals, large domestic or feral mammals like cattle, deer and pigs, could have an increased carrier rate after months of humid conditions. Therefore, large mammals probably also contribute to the transmission over consecutive years. The hypothesis of an increased capacity of the animal reservoir supported by both rodents and large Mammals is notably supported by a significant one-year lag impact of the incidence, a duration not explained by the abundance of short-lived rodents.

We found the strongest link between SST anomalies and leptospirosis cases, but we also found that variations in SST influenced rainfall and temperature, which in turn were directly associated with the risk for leptospirosis. Pathogenic *Leptospira* typically survive in wet soils, and heavy rain storms can flush the bacteria out into the water bodies where they can more readily infect humans. Therefore, there is a plausible causal link between that the changes in SST influence rainfall and leptospirosis cases. More generally, ENSO has a major impact on rainfall inter-annual variability in many regions in the world.

The results of this study should be evaluated in other countries in the Pacific, South Asia, Latin America, or Eastern Australia. New Caledonia is unique in its island geography, and New Caledonia's weather variability is highly impacted by ENSO. Therefore, other studies will need to determine whether SST anomalies or other ENSO-related parameters are good predictors of rainfall and leptospirosis cases in other, inland regions or island territories. In large countries like Thailand, there is evidence to suggest that different models should be used for different regions [46], making country-wide preparedness difficult. In the smaller Reunion Island [47], a season and meteorological-based model provides a good description of the incidence of the disease and might be used for prediction with a satisfactory accuracy.

Our study has limitations. Meteorological data were available from several weather stations around New Caledonia, but it was uncertain which station would best reflect the exposures of the population. We therefore evaluated several stations chosen in areas of highest leptospirosis incidence [23]. This is an ecological study, and we did not directly measure the impact of SST or rainfall on rodent density or *Leptospira* exposure in the environment. However, this link is well-established, and there is strong plausibility for a meteorological link for leptospirosis. The choice of an epidemic threshold is an arbitrary choice that should be driven by public health necessity. We used a seasonal “Serfling” baseline with a 95% confidence interval, which is commonly used for detecting influenza outbreaks. More or less conservative baselines could also be appropriate depending on the objective.

In these exploratory analyses, we considered many different El Niño and meteorological variables, with various lags. To reduce the number of predictors and the impacts of collinearity, we performed principal components analysis priori to multivariate model averaging [35]. While this analysis clearly established a strong correlation between the climate/meteorological variables and leptospirosis cases, the results should be interpreted cautiously. Since rainfall and temperature are likely on the causal pathway between El Niño and leptospirosis, including the meteorological variables in the multivariate model dampens the association between El Niño and leptospirosis.

The “forecasting” analysis was performed after SST anomaly was identified as being important in the full dataset. Additional years of data will be required to confirm the usefulness of this model in forecasting epidemics in New Caledonia.

Predicting outbreaks of leptospirosis is of prime importance for public health decision makers. Predictive models for leptospirosis are scarce. In our study, we demonstrate that the SST anomaly in the Niño Box 4 would allow a good prediction of leptospirosis incidence in New Caledonia 4 months in advance. This lag could allow increasing awareness and preparedness at all levels, from the general population to health practitioners. The use of mass media (radio or TV spots, advertising posters) for general population could allow reminding of risk and exposition factors as well as symptoms and encourage to promptly seeking medical advice. Likewise, medical professional forums and meetings could be used to increase awareness in the medical community. Because some at-risk occupations are vaccinated in New Caledonia, booster injections could also be planned and administered as part of this increased preparedness. Because particularly timely, it would probably additionally allow implementing practical field prevention measures, like rodent control operations or the cleaning out of river banks and sewage systems to minimize flooding risks, targeting areas of highest incidence.

## Supporting Information

Figure S1 Incidence of leptospirosis during the epidemic year 2008 in New Caledonia and location of the meteorological stations used in this study.
(PDF)

Table S1 Principal component analysis loadings.
(CSV)

Table S2 Correlation between El Niño indices and rainfall and temperature data from the same month in three locations in New Caledonia.
(PDF)

Table S3 Association between monthly leptospirosis cases and El Niño and meteorological variables, controlling for 12-month harmonic variation. Complete results.
(XLS)
