# Supplementary material for: El Niño Southern Oscillation and Leptospirosis Outbreaks in New Caledonia
Source: PLoS Negl Trop Dis. 2014 Apr 17;8(4):e2798. doi: 10.1371/journal.pntd.0002798 (PMC3990495; doi:10.1371/journal.pntd.0002798)
Supplement: Figure S1 — Incidence of leptospirosis during the epidemic year 2008 in New Caledonia and location of the meteorological stations used in this study. (PDF) [file pntd.0002798.s001.pdf]

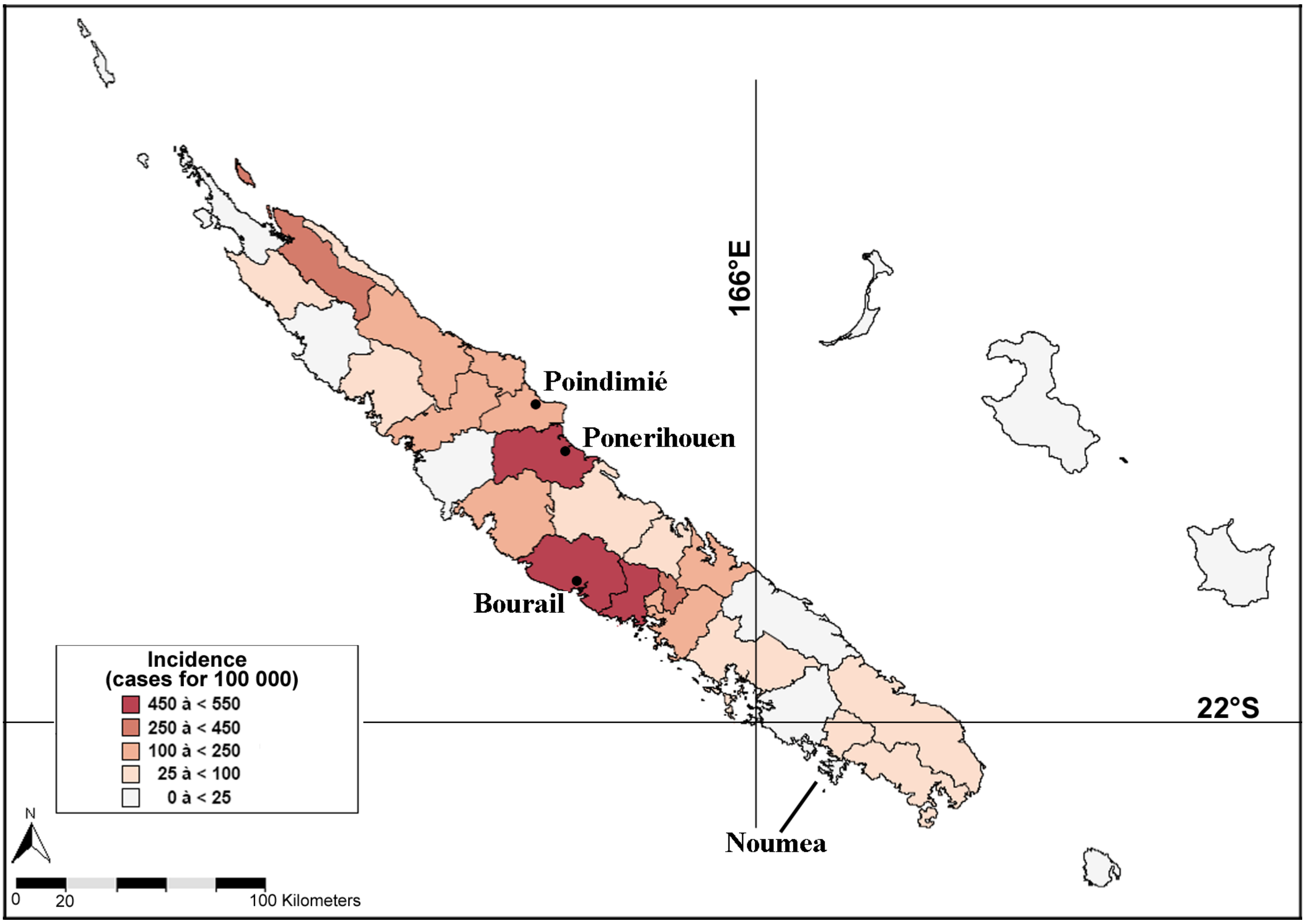

Figure S1: Incidence of leptospirosis during the epidemic year 2008 in New Caledonia and location of the meteorological stations used in this study.
