# Supplementary material for: El Niño Southern Oscillation and Leptospirosis Outbreaks in New Caledonia
Source: PLoS Negl Trop Dis. 2014 Apr 17;8(4):e2798. doi: 10.1371/journal.pntd.0002798 (PMC3990495; doi:10.1371/journal.pntd.0002798)
Supplement: Table S2 — Correlation between El Niño indices and rainfall and temperature data from the same month in three locations in New Caledonia. (PDF) [file pntd.0002798.s003.pdf]

Table S2. Correlation between El Niño indices and rainfall and temperature data from the same month in three locations in New Caledonia.

	Sea surface temperature anomaly (Box 4)	Oceanic Niño Index (ONI)
Rainfall		
Ponerihouen	-0.30 (-0.44, -0.15)	-0.32 (-0.44, -0.15)
Poindimie	-0.35 (-0.48, -0.21)	-0.37 (-0.48, -0.20)
Bourail	-0.22 (-0.37, -0.07)	-0.23 (-0.37, -0.07)
Mean maximum temperature		
Ponerihouen	-0.47 (-0.58, -0.34)	-0.52 (-045, -0.16)
Poindimie	-0.43 (-0.55, -0.29)	-0.51 (-0.58, -0.33)
Bourail	-0.31 (-0.46, -0.16)	-0.32 (-0.44, -0.16)

Values are Pearson partial correlation coefficient with 95% confidence intervals (Fisher), estimated when controlling for sine and cosine terms with a 12-month period. Correlation between ONI and sea surface temperature anomaly is 0.96.
